# Predicting protein–protein interactions between banana and *Fusarium oxysporum* f. sp. cubense race 4 integrating sequence and domain homologous alignment and neural network verification

**DOI:** 10.1186/s12953-022-00186-2

**Published:** 2022-03-29

**Authors:** Hui Fang, Cheng Zhong, Chunyan Tang

**Affiliations:** 1 Medical College, State Key Laboratory for Conservation and Utilization of Subtropical Agro-Bioresources, Guangxi University, Nanning, 530004 Guangxi China; 2 School of Computer, Electronics and Information, Guangxi University, Nanning, 530004 Guangxi China; 3 Guangxi Crop Genetic Improvement and Biotechnology Laboratory, Guangxi Academy of Agricultural Sciences, Nanning, 530007 Guangxi China

**Keywords:** Protein–protein interactions, Banana, *Fusarium oxysporum* f. sp. cubense race 4, Sequence alignment, Prediction

## Abstract

**Background:**

The pathogen of banana *Fusarium oxysporum* f. sp. cubense race 4(Foc4) infects almost all banana species, and it is the most destructive. The molecular mechanism of the interactions between *Fusarium oxysporum* and banana still needs to be further investigated.

**Methods:**

We use both the interolog and domain-domain method to predict the protein–protein interactions (PPIs) between banana and Foc4. The predicted protein interaction sequences are encoded by the conjoint triad and autocovariance method respectively to obtain continuous and discontinuous information of protein sequences. This information is used as the input data of the neural network model. The Long Short-Term Memory (LSTM) neural network five-fold cross-validation and independent test methods are used to verify the predicted protein interaction sequences. To further confirm the PPIs between banana and Foc4, the GO (Gene Ontology) and KEGG (Kyoto Encylopedia of Genes and Genomics) functional annotation and interaction network analysis are carried out.

**Results:**

The experimental results show that the PPIs for banana and foc4 predicted by our proposed method may interact with each other in terms of sequence structure, GO and KEGG functional annotation, and Foc4 protein plays a more active role in the process of Foc4 infecting banana.

**Conclusions:**

This study obtained the PPIs between banana and Foc4 by using computing means for the first time, which will provide data support for molecular biology experiments.

**Supplementary Information:**

The online version contains supplementary material available at 10.1186/s12953-022-00186-2.

## Introduction

Banana (Musaspp.) is a monocotyledonous perennial plant of the Musa genus in Musaceae. Banana is the largest herbaceous flowering plant in the world, and its fruit is edible. Banana grows in tropical and subtropical regions and is the fourth largest food crop after rice, wheat, and corn in some countries and regions [1]. Banana *Fusarium oxysporum* f. sp. cubense race 4(Foc4), also known as yellow leaf disease and Panama disease, is a typical fungal soil-borne disease caused by *Fusarium oxysporum* f.sp.cubense infection, which destroys banana vascular bundles and causes plant death [2]. Foc4, the pathogen of banana *Fusarium oxysporum* f. sp. cubense race 4, infects almost all banana species, and it is the most destructive [3]. The pathogenic process of *Fusarium oxysporum* needs to go through the identification process between pathogen and banana root. The pathogen reaches and adheres to the surface of the banana root, and *Fusarium oxysporum* produces a series of pathogenic factors, such as secreted effector protein factors [4], pathogenicity-related enzymes [5], and toxins [6]. The pathogen invades the inside of the host, colonizes in banana, and shows the symptoms on the outside [7]. At present, some progress have been achieved in the research of banana Foc4. Some pathogenic factors, cell wall degrading enzymes, and toxins of banana Foc4 have been found. Meanwhile, some banana resistance genes, active substances and hormones related to resistance have been discovered through transcriptomics and proteomics. However, up to now, there are no effective measures to control banana Foc4, and its pathogenic mechanism is not completely clear. Therefore, the molecular mechanism of the interactions between *Fusarium oxysporum* and banana still needs to be further investigated.

When the pathogen's proteins invade plants, the plants start the host's defense response to the invaded pathogens. Protein–protein interactions(PPIs) between plant protein and pathogenic protein are crucial to studying the molecular basis of pathogenesis [8]. The PPI analysis methods can be divided into biological experiment-based methods and bioinformatics-based methods. The biological experiment-based methods mainly include yeast two-hybrid [9], bimolecular fluorescence complementation [10], and immunoprecipitation [11]. The biological experiment-based methods have some disadvantages, such as time-consuming, high cost, and low coverage. The bioinformatics-based methods have the advantages of high efficiency and low cost, and they have the disadvantage of the existence of false positives. With the rapid development of omics data, the biological experiment-based methods are difficult to meet the requirement of high-throughput biological data. At present, the public databases DIP [12], HPRD [13], BioGRID [14], IntAct [15], MINT [16], and HPIDB [17] store a large number of experimentally verified PPIs data, which provide data sources for predicting PPIs using bioinformatics methods. The interolog method and domain-domain method have been used to predict PPIs in some fields. Recently, some researchers used these two methods to predict the intraspecific PPIs among bacterial blight pathogen, rice, corn, and cassava [18–21], and FWHT-RF [22] can be a useful supplementary method to predict potential PPIs in plants.

Interspecies PPI has been reported in the study of human and pathogenic bacteria, which is used to predict the PPIs between human and hepatitis C virus [23], between humans and Bacillus anthracis [24], and between humans and Plasmodium falciparum [25]. For the study of PPIs between plant and pathogen, Li et al. predicted 3074 protein interactions between Arabidopsis thaliana and Ralstoniasolanacearum on the database DIP by the interolog method and domain-domain method. These protein interactions include 119 Ralstoniasolanacearum proteins and 1442 Arabidopsis thaliana proteins. The data set of PPIs was verified by GO functional annotation and network characteristic analysis [26].

By using the interolog method and domain-domain method, Ma et al. predicted 523 PPIs between rice and Magnaporthegrisea, including 27 rice blast proteins and 236 rice proteins [27]. The obtained PPI data set was verified by the machine learning method, and the protein function was analyzed by GO and the KEGG pathway. Zheng et al. [28] used the structure-based method and generated a global PPI network consisting of 2,018 PPIs involving 1,344 rice and 418 blast fungus proteins. To our knowledge, the research on predicting PPIs between plants and pathogens has only been reported on the model plants Arabidopsis thaliana, rice, and their pathogens. But there are no related reports on predicting PPIs between banana and Foc4 based on the Bioinformatics methods. The study on the interactions between banana and Foc4 has been mainly conducted from the independent perspective of infection of Foc4 pathogenic factors and active substances related to banana resistance. The genes or proteins differentially expressed in bananas could be obtained in previous studies, but the effectors of Foc4 interacting with banana protein could not be identified.

This paper has the following contributions. We proposed a computing method for predicting PPIs for banana and Foc4 for the first time. We encoded the predicted PPIs sequences for banana and Foc4 by the conjoint triad method and autocovariance method respectively to obtain continuous and discontinuous information of protein sequences, verify the predicted PPIs may interact in sequence structural characteristics by the The Long Short-Term Memory (LSTM) neural network five-fold cross-validation and independent test methods [29], and further functionally verify the PPIs by the GO, KEGG function annotation, and interaction network analysis. The predicted PPIs between banana and Foc4 will provide data support for molecular biology experiments.

## Materials and methods

### Datasets

We first downloaded 45,856 banana proteins in banana protein sequences from https://banana-genome-hub.southgreen.fr and 14,459 Foc4 protein sequences from ftp://ftp.ncbi.nlm.nih.gov/genomes/all/GCA/000/350/365/GCA_000350365.1_Foc4_1.0, respectively. Secondly, We downloaded all PPIs of six model species, Arabidopsis thaliana, nematode, Drosophila, yeast, Escherichia coli, and human, from the database MINTat https://mint.bio.uniroma2.it/, the database DIP at https://dip.doe-mbi.ucla.edu/dip/main.cgi, the database TAIR at https://www.arabidopsis.org/, the database BioGRID at https://downloads.thebioged.org/biogerid/release-archive/ biogerid-3.5.166/, and the database INTACT at https://www.ebi.ac.uk/intact/, respectively. Thirdly, we downloaded 118,921 PPIs from the database MINT, 76,881 PPIs from the database DIP, 2656 PPIs from the database TAIR, and 183,768 PPIs from the database IntAct. Finally, we downloaded 62,782 pathogen-host interspecific protein interactions from the databaseHPIDB at http://hpidb.igbb.msstate.edu/.All domain-domain interaction template PPIs were downloaded from the database3DID [30] at https://3did.irbbarcelona.org/. The corresponding protein sequences of the above six species were downloaded from the database Uniprot at https://www.uniprot.org/. Different databases may use different IDs for the same protein. We used the software tool Biomart [31] to convert the different protein IDs into uniform IDs.

### Methods

**Fig. 1:**
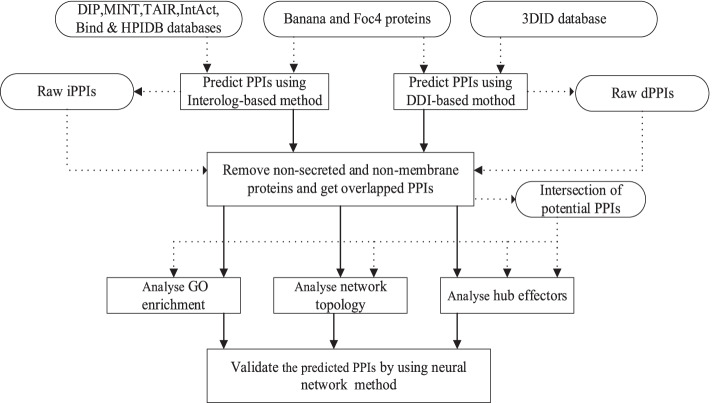
Process of predicting PPIs between banana and Foc4, where the solid arrow represents ‘control flow direction’ and the dashed arrow denotes ‘data flow direction’

We first downloaded the experimentally verified intra-species and inter-species PPIs from the database as the interaction template. Next, we applied the interolog method and domain-domain method to predict the data sets of PPIs between banana and Foc4 to find the common PPIs between banana and Foc4. Thirdly, we used the conjoint triad(CT) [32] and auto covariance(AC) [33] to code protein sequence features to obtain the structure information of continuous and discontinuous protein sequences. Fourthly, we verified the predicted PPIs data sets for banana and Foc4 by using LSTM neural network five-fold cross-validation method and independent test method. Finally, we computed the accuracy, sensitivity, specificity, receiver operating characteristic curve (ROC), and area under the curve(AUC) of the predicted results. Figure 1 shows the process of predicting PPIs between banana and Foc4, in which iPPIs indicate interolog PPIs, dPPIs represent domain-domain PPIs, and DDI denotes domain-domain interactions.

### Predicting PPIs between banana and Foc4

The interolog method is a means for predicting homologous interactions. Its main idea is that homologous proteins may have similar properties. If two proteins A and B interact with each other via verified experiments, and two proteins A' and B' are homologous proteins of A and B respectively, then according to the principle that homologous proteins have similar properties, proteins A' and B' may also interact with each other [23]. The idea of the domain-domain interaction prediction method is that if proteins C and D contain domains C and D which can interact with each other, proteins C and D may interact with each other [24].

Based on the protein sequence data of banana and Foc4, we used the interolog method and domain-domain method to predict the interactions between banana and Foc4. We selected the transmembrane or secreted proteins in Foc4 as the protein infecting banana [26] and obtained the final PPIs data set between banana and Foc4.

For the interolog method, we used the local sequence alignment tool BLAST to find the homology proteins, where the parameter *E* was set to 0.00001, the sequence identity was set to 30%, and the coverage was set to 80%[26, 27]. Firstly, the protein sequences of six model species are compared with banana and Foc4 to find out the orthologous proteins between banana and Foc4. Then, the host protein sequences in the database HPIDB are compared with the banana protein sequences and the pathogen protein sequences are compared with the Foc4 protein sequences to obtain interspecific homologous proteins.

We submitted the protein sequences of banana and Foc4 to the database 3DID to find out the domains contained in each protein, where the value of parameter *E* was set to 0.00001 and the sequence identity was set to 90% [26]. If any PPI of banana and Foc4 contains a couple of interactive domains in the database 3DID, it is considered that this pair of proteins for banana and Foc4 may interact with each other [34].

We applied the two software tools signalP [35] and WoLFPSOFT [36] with the default values of their parameters to find secretory proteins. If a protein predicted by signalP contains a signal peptide and is located as extracellular by WoLFPSOFT, the protein is a secretory protein. In addition, we used the software TMHMM2.0 [37] to predict transmembrane proteins in Foc4 proteins. If the number of transmembrane helices predicted by TMHMM is greater than 1, the proteins are considered to be transmembrane proteins [38].

### PPIs coding of sequence features

Proteins are biomolecules composed of amino acids, while protein sequences are represented by 20 standard amino acids. Encoding the sequence feature of a protein is to extract the feature vector from the protein sequence. The sequence feature extraction transforms the original sequences into a fixed-length numerical vector. In recent years, some researchers have proposed some methods to predict PPIs using only protein sequence information, but these methods can not fully capture interaction information from continuous and discontinuous amino acid fragments at the same time.

In order to solve the above problem, the conjoint triad (CT) method and auto covariance(AC) method were used to encode sequence features. By using the CT method, 20 amino acids are divided into seven categories according to the volume of even electrodes and side chain volume. Each three consecutive amino acids is regarded as a basic unit, and the class frequency of all basic units in a protein is counted. The AC method mainly considers the proximity effect and uses both the continuous and discontinuous sequence information in a protein sequence. The number of all possible kinds for each basic unit is 7 × 7 × 7 = 343. Thus, the final feature vector with 686-dimension contains the features of two proteins interacting with each other. Min–max normalization was performed on the feature vectors to map the result of encoding each protein pair into the interval [0,1], so as to remove the influence of protein length on frequency counting. Let ${f_i}$ represent the *i*-th component of a protein eigenvector, the *i*-th component of a normalized protein feature vector, *d*_*i*_, is computed as follows [32]:

1 $$d_{i} = \frac{{f_{i} - \min \{ f_{1} ,f_{2} ,......,f_{343} \} }}{{\max \{ f_{1} ,f_{2} ,......,f_{343} \} }},i = 1,2,3, \ldots ,343$$

The interactions between amino acids are reflected by seven physical and chemical characteristics of amino acids. The seven physical and chemical properties are hydrophobicity, hydrophilicity, net charge index, polarity, polarizability, solvent accessible surface area, and side chain volume, respectively. Each protein sequence is transformed into a 7-dimensional vector, and each amino acid is represented by a normalized value of seven descriptors. The initial values of seven physical and chemical properties of 20 amino acids can be found in [33]. The variance $A{C_{lag,j}}$ is computed as follows [33]:

2 $$A{C_{lag,j}} = \frac{1}{n - lag}\sum\limits_{i = 1}^{n - lag} {({X_{i,j}} - \frac{1}{n}\sum\limits_{i = 1}^n {{X_{i,j}}} )({X_{(i + lag),j}} - \frac{1}{n}\sum\limits_{i = 1}^n {{X_{i,j}}} )}$$

where *lag* represents the distance between the two amino acid residues, *n* is the length of protein sequence *X*, *X*_*i*,*j*_ represents the *j*-th descriptor in the *i*-th position of a protein sequence. In this paper, seven physical and chemical properties are used and the optimal value of *lag* is set to 30 [39]. After AC transformation, each protein sequence has been transformed into a 210-dimensional vector. Combined with the CT method, each PPI sequence has been transformed into a vector of (343 + 210) × 2 = 1106 dimensions.

### Verification

We used the interolog method and domain-domain method to deal with the proteins of banana and Foc4 to obtain their PPIs, and treated these PPIs as the positive samples with size 739. We verified the predicted results by the five-fold cross-validation method and independent test method, respectively. The Long Short-Term Memory(LSTM) neural network [40] was used to predict PPIs between banana and Foc4.

By using the characteristic coding of the PPIs between banana and Foc4, the original protein sequence was converted into a fixed-length numerical vector which was used as the input of the LSTM neural network. The input layer of LSTM neural network was a feature vector composed of the forward and backward hidden layer output vectors *h*_*f*_ and *h*_*b*_. The corrected linear unit(relu) was used as the activation function in the hidden layer, and the softmax function was used in the output layer. According to the results of the CT and AC coding schemes, the input sequence was $X = \left( {{x_1},{x_2},{x_3},...,{x_{1106}}} \right)$ and the prediction model outputs a corresponding result sequence was $Y = \left\{ {{y_1},{y_2},{y_3},...,{y_{1106}}} \right\}.$ In the prediction model, the learning rate was set to 0.001, the batch size was 128, and the fully connected layer has 128 neurons. In five-fold cross-validation, we randomly selected negative samples from banana and Foc4 proteome. The size of the selected negative samples was the same as the size of the predicted PPIs.. The selected negative samples filtered out the samples in the predicted PPIs between banana and FOC with a sequence consistency greater than 20%. When the size of positive samples is *m*, the size of negative samples is 10 × *m*. We selected the samples with size of 2 × *m*/3 in the positive samples and the samples with size of 2 × *m*/3 in the negative samples to form the training set, and selected the remaining positive samples with size of *m*/3 and the remaining negative samples with size of 10 × *m*-2 × *m*/3 = 28 × *m*/3 to form the test set.

In this paper, we used the accuracy *ACC*, sensitivity *Sn*, specificity *Sp*, receiver operating characteristic curve *ROC*, and area under curve *AUC* to evaluate the prediction effect [23]:

3 $$ACC = \frac{TN + TP}{{TN + TP + FN + FP}}$$

4 $$S{\text{n}} = \frac{TP}{{TP + FN}}$$

5 $$Sp = \frac{TN}{{TN + FP}}$$

where *TN* is the number of true counterexamples, *TP* represents the number of true examples, *FN* denotes the number of false counterexamples, and *FP* is the number of false-positive examples.

Each protein is used as a node and the interaction between each pair of proteins is represented as an edge, a PPIs network is created by all the nodes and edges. We used the software Cytoscape3.7 [41] to visualize the PPIs network to conveniently and intuitively observe the characteristics of the network. We used the ClusterViz plug-in in Cytoscape [41] to divide the interaction network into different functional modules. We executed the algorithm ClusterVizuse FAG-EC [42] to partition the network into several subnetworks. The median centrality V_*i*_ of node *i* in the network is calculated as follows:

6 $${v_i} = \sum\limits_{s \ne t \ne i} {\frac{{n_{st}^i}}{{{g_{st}}}}}$$

where ${g_{st}}$ denotes the number of the shortest paths from node *s* to node *t*, and $n_{st}^i$ represents the number of the shortest paths from node *s* to node* t* via node *i* in the network.

We applied the software TBTools [43] to carry out the GO (Gene Ontology) functional enrichment analysis of PPIs. According to the specification for TBTools, we set the value of parameter *p* < 0.05 and used Bonferroni correction [44]. KEGG (Kyoto Encylopedia of Genes and Genomics) enrichment analysis (*p*-value < 0.05) of PPIs was performed by using KOBAS2.

#### Results

### Experimental environment

The computer used was with Intel (R) Xeon (R) W-2133 CPU @ 3.6 GHz processor and memory capacity 8 GB running operating system Windows10. The prediction algorithm was implemented by Python3 programming.

### Experimental results

We first predicted 26,910 PPIs and 376,755 PPIs between banana and Foc4 by using the interolog method and domain-domain method, respectively. Table 1 shows the results of predicted PPIs, where 739 interactions with 515 banana proteins and 81 Foc4 proteins are common overlapping PPIs predicted by the interolog method and domain-domain method. Method1 represents the interolog method, and Method2 denotes the domain-domain method. The detailed data sets of all predicted results are given in Supplementary table 1.

**Table 1:** Statistical information of predicted PPIs between banana and Foc4

Prediction method	Number of PPIs	Number of Banana proteins	Number of Foc4 proteins
Method1	26,910	5938	697
Method2	376,755	18,965	1916
Common parts of predicted results of Method1 and Method2	739	515	81

It can be seen from the results in Table 1 that the number of PPIs predicted by the interolog method is less than that of PPIs predicted by the domain-domain method. This is because the interolog method adopts the homologous sequence-based alignment, which depends on the amount of data in the existing database, while the domain-domain method is based on the interactive domains contained in proteins, and a protein can contain two or more interactive domains [45].

We extracted the feature vector of proteins in banana-Foc4 PPIs, and analyzed the reliability of banana-Foc4 PPIs predicted by the LSTM neural network-based five-fold cross-validation method and independent test method. Table 2 shows the results of sensitivity *Sn*, specificity *Sp*, accuracy *ACC*, and receiver operating characteristic curve *ROC* of the predicted banana-Foc4 PPIs.

**Table 2:** Values of *Sn*, *Sp*, *ACC*, *ROC*, and running time of predicted banana-Foc4 PPIs

Model	Test method	*Sn(%)*	*Sp(%)*	*ACC(%)*	*ROC*	Time(s)
LSTM	five-fold cross validation	90.75	98.52	94.45	0.94	54.37
	independent test	85.81	92.85	89.78	0.87	58.63
SVM	five-fold cross validation	88.85	88.14	94.45	0.94	0.74
	independent test	79.07	84.56	84.23	0.85	12.54

We can see from Table 2 that for the LSTM model, the results predicted by the five-fold cross-validation method were better than the ones predicted by the independent test method, and the results predicted by the LSTM model were better than the ones predicted by the SVM (Support Vector Machine) model, while the LSTM model required much longer computational time than the SVM model. On the other hand, the experimental results also show that the PPIs between banana and Foc4 predicted by five-fold cross-validation and independent test methods have high structural similarity. It illustrates that the PPIs between banana and Foc4 may interact in sequence structure characteristics.

**Fig. 2:**
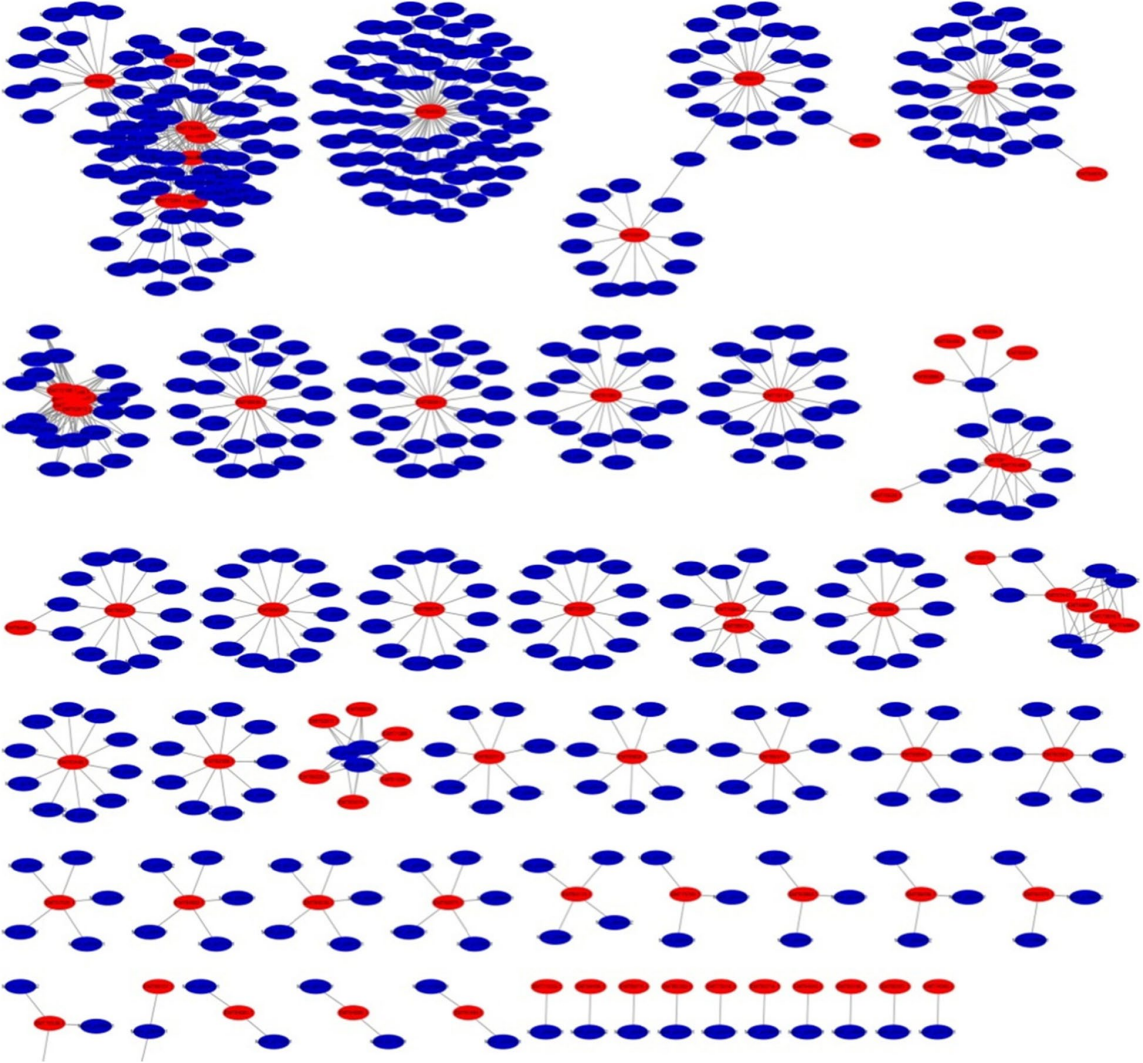
PPIs network between banana and Foc4, where the red node represents Foc4 protein, and the blue node denotes banana protein

The following is to analyze the network structure characteristics of the PPIs between banana and Foc4 predicted by the experiment. By using Cytoscape, each protein in the interactions between banana and Foc4 was treated as a node, and each interaction between banana and Foc4 was treated as an edge. The result of the PPIs network between banana and Foc4 is shown in Fig. 2, and the detailed information of the PPIs network is given in Supplementary table 2.

In the PPI network, the connectivity of a protein is defined as the number of all other proteins linking to this protein. The connectivity is an index of evaluating the importance of a protein in the network. From Fig. 2 we can see that the average connectivity of Foc4 protein was 9.12 and the average connectivity of banana protein was 1.43. This indicates that the connectivity of Foc4 protein was higher than that of banana protein in the PPI network for banana and Foc4, and Foc4 protein played a more active role, which affected a series of biological processes of banana infected by Foc4. It can also be seen from Fig. 2 that the PPI network for banana and Foc4 was divided into 51 sub-networks, in which the largest sub-network contains 86 nodes, the smallest sub-network has only two nodes, and there are 30 sub-networks with more at least to 6 nodes. Some complex sub-networks with more nodes contain multiple Foc4 proteins. Some sub-networks only contain one Foc4 protein. The smallest sub-network only has one banana interacting with the Foc4 protein. In addition, we found that three proteins of Foc4, namely EMT64532.1, EMT73264.1, and EMT73245.1, interact with 72, 58, and 29 proteins of banana, respectively. This illustrates that these three proteins of Foc4 play important roles in the interactions, and these results will provide a basis for future biological experiments.

To annotate the GO function of PPIs for banana and Foc4, we first aligned the banana protein with SwissProt protein by the software BLAST. Then, we compared the obtained Foc4 protein with SwissProt protein. Finally, we used the TBTools to annotate the GO function PPIs for banana and Foc4. The top 20 annotated results of proteins for Foc4 are shown in Table 3, and the annotated results of proteins for banana are shown in Table 4.

**Table 3:** Top 20 GO annotated results of proteins for Foc4

GO Name in Biological Process	GO ID	*P*_value	Hit Counts
membrane fusion	GO:0,061,025	6.87E-08	8
export from cell	GO:0,140,352	1.49E-07	17
transport	GO:0,006,810	2.63E-07	47
establishment of localization	GO:0,051,234	4.96E-07	47
vesicle fusion	GO:0,006,906	8.42E-07	6
export across plasma membrane	GO:0,140,115	8.91E-07	8
localization	GO:0,051,179	1.56E-06	49
organelle membrane fusion	GO:0,090,174	2.50E-06	6
vesicle organization	GO:0,016,050	3.15E-06	9
organelle fusion	GO:0,048,284	3.18E-06	8
transmembrane transport	GO:0,055,085	4.18E-06	28
membrane organization	GO:0,061,024	4.82E-06	15
xenobiotic detoxification by transmembrane export across the plasma membrane	GO:1,990,961	7.81E-06	6
xenobiotic transport	GO:0,042,908	9.77E-06	6
intracellular transport	GO:0,046,907	2.64E-05	21
organophosphate ester transport	GO:0,015,748	2.67E-05	7
cellular localization	GO:0,051,641	3.48E-05	27
organic substance transport	GO:0,071,702	4.36E-05	33
mitochondrial transport	GO:0,006,839	8.27E-05	8
establishment of localization in cell	GO:0,051,649	1.00E-04	22

**Table 4:** GO Annotated results of proteins for banana

GO Name in Biological Process	GO ID	*P*_value	Hit Counts
transport	GO:0,006,810	3.33E-16	262
translation	GO:0,006,412	3.53E-10	63
catabolic process	GO:0,009,056	1.54E-05	129
protein metabolic process	GO:0,019,538	2.48E-05	194
tropism	GO:0,009,606	1.45E-04	20
cellular homeostasis	GO:0,019,725	5.13E-04	34
embryo development	GO:0,009,790	1.15E-02	61
cellular component organization	GO:0,016,043	1.16E-02	206
cell–cell signaling	GO:0,007,267	2.25E-02	21

It can be seen from Table 3 that in the annotated GO function results of Foc4 protein, the top three ones are membrane fusion, export from cell, and transport respectively. In addition, we can also see that Foc4 protein annotates vesicle fusion, export across membrane, transmembrane transport, and membrane organization, which are all related to cell membrane function. Foc4 protein must cross the cell membrane if it wanted to enter banana and interact with banana protein.

Table 4 shows that in the annotated GO function results of banana proteins, the top three ones are transport, translation, and catabolic process respectively. Some banana R-proteins(resistance proteins) are annotated with tropism, cellular homeostasis, cell–cell signaling, and other functions, all of which are related to the response of cells to external stress. Foc4 protein enters the banana, and the banana uses the specificity of intracellular resistance proteins to recognize the effector and trigger immune response [46].

It can be seen from Table 5 that in the annotated KEGG function results of Foc4 protein, there are many protein annotates membrane transport, ABC transporters, interactions in vesicular transport and transporters, which are all related to the environmental information processing pathway. The annotated KEGG function results of banana protein in Table 6, there are many protein annotates interactions in vesicular transport, membrane transport, ABC transporters, which are related to the environmental information processing pathway.

**Table 5:** KEGG Annotated results of proteins for Foc4

Pathway	*p*-value	hits
membrane transport	4.02E-07	7
ABC transporters	4.02E-07	7
interactions in vesicular transport	6.88E-07	5
environmental Information Processing	6.24E-05	7
signaling and cellular processes	8.55E-04	22
transporters	1.91E-03	14
folding, sorting and degradation	2.29E-03	10
Ribosome	5.68E-03	6
chaperones and folding catalysts	1.32E-02	5
genetic Information Processing	1.48E-02	18
enzymes with EC numbers	3.80E-02	6

**Table 6:** KEGG Annotated results of proteins for banana

Pathway	*p*-value	Hits
ubiquitin mediated proteolysis	0	67
interactions in vesicular transport	0	38
Ribosome	0	55
protein processing in endoplasmic reticulum	2.22E-16	56
folding, sorting and degradation	4.44E-16	126
genetic Information Processing	5.55E-16	191
membrane transport	2.19E-10	12
ABC transporters	2.19E-10	12
translation	1.18E-07	63
ribosome biogenesis in eukaryotes	1.98E-02	8

The GO annotation results of predicted PPIs between banana and Foc4 show that Foc4 protein were annotated the functions related to cell membrane such as vesicle fusion, transmembrane export, transmembrane transport and membrane tissue, and banana protein were annotated the functions related to external stress response such as transport, tropism, cell automatic regulation and cell signal transduction. The KEGG annotation results show that the Foc4 protein annotates membrane transport, ABC transporters, interactions in vesicular transport and transporters. The banana protein were annotated the functions related to the environmental information processing pathway. This illustrates that the PPIs between banana and Foc4 predicted by our method are reliable from the perspective of GO and KEGG functional annotation.

## Discussion

One of the characteristics of this study is that the intra-species and inter-species PPIs downloaded from the database were used as interaction templates, the PPIs between banana and Foc4 were predicted by the interolog method and the domain-domain method respectively, and the intersection of PPIs predicted by these two methods was taken as the final predicted result which was more accurate. In addition, the problem studied here is inter-species protein interaction, which uses not only intra-species protein interaction of model species as prediction template but also uses inter-species protein interaction of multiple species as prediction template. The template of interspecific interaction prediction comes from the database HPIDB, which contains PPIs of 66 species of animals, plants, and many pathogens, including interspecific protein interactions between animals and microorganisms and the ones between plants and microorganisms.

In this paper, we coded the sequence of PPIs by the combined use of CT method and AC method. The CT method regards every three consecutive amino acids as a basic unit and counts the class frequency of all basic units in protein, while the AC method mainly pays close attention to the proximity effect. In this way, the continuous and discontinuous sequence information of proteins can be used at the same time, which makes the prediction result more accurate. We verified the PPIs dataset between banana and Foc4 by LSTM neural network-based five-fold cross-validation method and independent test method.

By observing the results of GO and KEGG function annotation and PPIs network analysis, we found that there were many Foc4 interacting with host protein in PPIs between banana and Foc4. In addition, we also discovered that many Foc4 protein GO annotations were related to vesicle fusion, export across membrane, transmembrane transport, and membrane organization. The Foc4 protein KEGG annotations were related transporters, environmental information processing, ABC transporters, and membrane transport pathway. This indicates that Foc4 protein needs to be secreted outside the cell and must cross the cell membrane in order to infect bananas. At the same time, we can see that in the predicted PPIs between banana and Foc4, the functions of proteins related to external stress, cellular homeostasis, and cell–cell signaling are enriched, and the pathogenic molecules in vitro are recognized by proteins in banana and a series of immune responses downstream are stimulated. Therefore, these enriched proteins may be involved in the identification of pathogenic proteins of Foc4. This illustrates that the PPIs between banana and Foc4 proteins predicted by our method are reliable from the perspective of GO and KEGG functional annotation.

## Conclusion

The innovation and characteristic of this paper is that both the interolog method and domain-domain method were applied to predict the PPIs between banana and Foc4, and the dataset of PPIs between banana and Foc4 was obtained by computing means for the first time. The combination of the CT and AC methods was used to encode protein characteristics to obtain the continuous and discontinuous sequence information of proteins. The predicted banana-Foc4 PPIs dataset was verified by LSTM neural network-based five-fold cross-validation method and independent test method. The GO, KEGG annotation, and interaction network analysis of banana and Foc4 protein interactions shows that there were indeed PPIs between banana and Foc4, and several Foc4 proteins interact with host protein together. The dataset of PPIs between banana and Foc4 predicted by computing method will provide a basis for the study of banana Fusarium wilt, and also offer a new means for analyzing the molecular mechanism of interactions between banana and Foc4. In the future, we will investigate the biological experiment method to verify whether there may be some false positives in the protein mutual network between banana and Foc4 constructed by the computation method.

## Supplementary Information

**Additional file 1:** **Supplementary table 1.** Predicted protein-protein interaction pairs between banana and Foc4.

**Additional file 2:** **Supplementary table 2.** The degrees of nodes.

## Data Availability

All data generated or analyzed during this study are included in this published. article and its supplementary information files.

## Abbreviations

GO: Gene Ontology
Foc4: *Fusarium oxysporum* f. sp. cubense race 4
PPIs: Protein–protein interactions
KEGG: Kyoto Encylopedia of Genes and Genomics
CT: Conjoint triad
AC: Auto covariance
LSTM: Long Short-Term Memory
ROC: Receiver operating characteristic curve
AUC: Area under the curve
iPPIs: Interolog Protein–protein interactions
dPPIs: Domain Protein–protein interactions
